# PEDOT:PSS-coated platinum electrodes for neural stimulation

**DOI:** 10.1063/5.0153094

**Published:** 2023-12-05

**Authors:** Gerwin Dijk, Jolien Pas, Katarina Markovic, Janez Scancar, Rodney Philip O'Connor

**Affiliations:** 1 Department of Bioelectronics, Mines Saint-Etienne, Centre CMP, Gardanne 13541, France; 2 Panaxium SAS, Aix-en-Provence 13100, France; 3 Department of Environmental Sciences, Jožef Stefan Institute, Jamova 39, 1000 Ljubljana, Slovenia; 4 Jožef Stefan International Postgraduate School, Jamova 39, 1000 Ljubljana, Slovenia; 5 Department of Chemical Engineering, Polytechnique Montreal, 2900 boul Edouard-Montpetit, Montreal H3T 1J4 Canada

## Abstract

Safe and long-term electrical stimulation of neurons requires charge injection without damaging the electrode and tissue. A common strategy to diminish adverse effects includes the modification of electrodes with materials that increases the charge injection capacity. Due to its high capacitance, the conducting polymer PEDOT:PSS is a promising coating material; however, the neural stimulation performance in terms of stability and safety remains largely unexplored. Here, PEDOT:PSS-coated platinum (Pt-PEDOT:PSS) microelectrodes are examined for neural stimulation and compared to bare platinum (Pt) electrodes. Microelectrodes in a bipolar configuration are used to deliver current-controlled, biphasic pulses with charge densities ranging from 64 to 255 *μ*C cm^−2^. Stimulation for 2 h deteriorates bare Pt electrodes through corrosion, whereas the PEDOT:PSS coating prevents dissolution of Pt and shows no degradation. Acute stimulation of primary cortical cells cultured as neurospheres shows similar dependency on charge density for Pt and Pt-PEDOT:PSS electrodes with a threshold of 127 *μ*C cm^−2^ and increased calcium response for higher charge densities. Continuous stimulation for 2 h results in higher levels of cell survival for Pt-PEDOT:PSS electrodes. Reduced cell survival on Pt electrodes is most profound for neurospheres in proximity of the electrodes. Extending the stimulation duration to 6 h increases cell death for both types of electrodes; however, neurospheres on Pt-PEDOT:PSS devices still show significant viability whereas stimulation is fatal for nearly all cells close to the Pt electrodes. This work demonstrates the protective properties of PEDOT:PSS that can be used as a promising approach to extend electrode lifetime and reduce cell damage for safe and long-term neural stimulation.

## I. INTRODUCTION

Modulation of the nervous system by electrical stimulation is a rapidly expanding field due to proven successes and promising new applications for the treatment of neurological diseases and disorders.^1–7^ Recent progress includes the use of microelectrodes for higher spatial resolution,^8,9^ thin film devices to minimize invasiveness,^10^ and emerging electrode materials to enhance the performance.^11^ Despite tremendous effort over multiple decades, one of the major challenges of electrical stimulation therapies remains the development of a stable neural interface that maintains its functionality throughout the duration of the treatment, which possibly spans the patient's lifetime.^12,13^

To achieve a long-term functional interface, the stimulation electrode must deliver sufficient charge to modify neural activity without causing any damage to the tissue or electrode.^14^ To minimize adverse effects, charge is ideally injected capacitively or through reversible faradaic processes without initiating irreversible faradaic reactions that can degrade the electrode and expose cells to toxic species.^15,16^ The electrode material plays a crucial role in the charging of the interface since its electrochemistry dictates the capacitive and faradaic mechanisms.

Platinum (Pt) is the most common electrode material for clinical applications and is likely the most investigated material in the context of neural stimulation. However, the use of Pt imposes some key limitations that restricts the functionality and applicability of such electrodes. First, Pt has a limited charge injection capacity which is challenging for small size electrodes and when higher charge densities are required.^17^ Second, Pt electrodes can cause stimulation-induced tissue damage as modeled by the Shannon equation which defines charge densities thresholds that are considered safe.^18,19^ Finally, stimulation pulses can lead to the dissolution of Pt, an irreversible process that results in the corrosion of the electrode and soluble Pt species in the biological medium.^20,21^ *In vitro*, the corrosion products of Pt electrodes have shown to induce cell death in a concentration- and cell type-dependent manner.^22,23^ *In vivo*, Pt-based cochlear implants corrode at relatively high charge densities without the loss of auditory neurons and neural function.^24^ Despite this apparent application dependency, Pt dissolution is undesired for any long-term neural interface because it limits the lifetime of the electrode, especially of thin film devices. The presence of proteins at the electrode interface and imbalanced waveforms have shown to reduce Pt corrosion; however, some dissolution persists.^21,25,26^ Park *et al.* demonstrated that graphene coatings suppress but do not completely prevent dissolution possibly because of defects and cracks in the material.^27^

To overcome these limitations, various material approaches have been proposed and the conducting polymer PEDOT:PSS has emerged as a promising candidate to enhance the electronic interface with living systems.^28–30^ PEDOT:PSS is commercially available, easy to process, noncytotoxic, and relatively stable in aqueous solutions.^31–33^ In addition to the electronic conductivity, PEDOT:PSS absorbs water and ions, which give rise to an interaction between the electrolyte and the polymer chains that takes place in the bulk of the material and facilitates an intimate connection with biological systems.^34^ It has been widely applied as a coating on metallic neural electrodes to increase the capacitance that greatly reduces the impedance and increases the charge injection capacity.^35,36^

The stability of PEDOT:PSS coatings is a matter of debate and depends on the underlying substrate material, processing, and the stimulation parameters. Severe cracking and delamination were reported for electrodeposited PEDOT:PSS on Pt electrodes after 2 weeks of stimulation.^37^ On the contrary, marginal changes in the impedance were reported after 7 weeks of continuous pulsing on PEDOT:PSS-coated gold electrodes.^38^ Chemically cross-linked PEDOT:PSS coatings on gold electrodes have shown delamination and corrosion of the underlying metal under accelerated aging and stimulation conditions.^39^ Despite these findings, PEDOT:PSS coatings on thin film Pt electrodes and the influence on metal dissolution and performance remain relatively unexplored.

Electrical stimulation with PEDOT:PSS has shown great promise for applications, such as neural stem cell differentiation^40^ and directing cell migration with electrotaxis,^41^ as well as modulation of nervous tissue.^42–44^ Despite encouraging developments, few studies report on microstimulation and the impact on cellular damage. Note that damage initiated by stimulation with PEDOT:PSS-coated microelectrodes are not included in the Shannon equation since this was derived from experiments with Pt electrodes. The high charge injection capacity of PEDOT:PSS is generally believed to reduce the risk of harmful electrochemical events. However, the charge injection mechanism of PEDOT:PSS is complex and the capacitive and faradaic processes occurring at the polymer–electrolyte interface are not fully understood yet.^34^ Recent reports have shown that stimulation with typical pulse parameters induces oxygen reduction reactions that lead to the formation of hydrogen peroxide which potentially affects the physiology of cells.^45^ Therefore, cell viability upon stimulation with PEDOT:PSS requires further investigation because this ultimately dictates its safety.

In this work, we examined and compared the stimulation performance of PEDOT:PSS-coated and bare Pt microelectrodes. PEDOT:PSS films were obtained through spin coating of a commercial dispersion, a deposition technique that easily integrates with other microfabrication processes. The experiments were divided into two parts. First, the electrode stability in phosphate buffered saline (PBS) was determined. We evaluated the change in physical appearance, the dissolution of Pt, and the stimulation performance. Second, the stimulation-induced response of clusters cortical cultures, also referred to as neurospheres, was assessed. Neurons are typically cultivated on planar devices with a 2D organization of cells to properly cover the entire surface. However, cell cultures that are grown in 3D clusters have demonstrated cell–cell interactions that better mimic the cytoarchitecture *in vivo*, with significant higher spontaneous activity, enhanced neurite outgrowth, and higher cell survival.^46–49^ Such 3D *in vitro* models provide excellent platforms to explore stimulation-induced activation and evaluate cell survival, which is a primary requirement for long-term functional stimulation. We verified the activation of these neurospheres through calcium imaging and investigated cell survival with live/dead assays after continuous electrical stimulation.

## II. RESULTS

### A. Electrode design

**FIG. 1.**
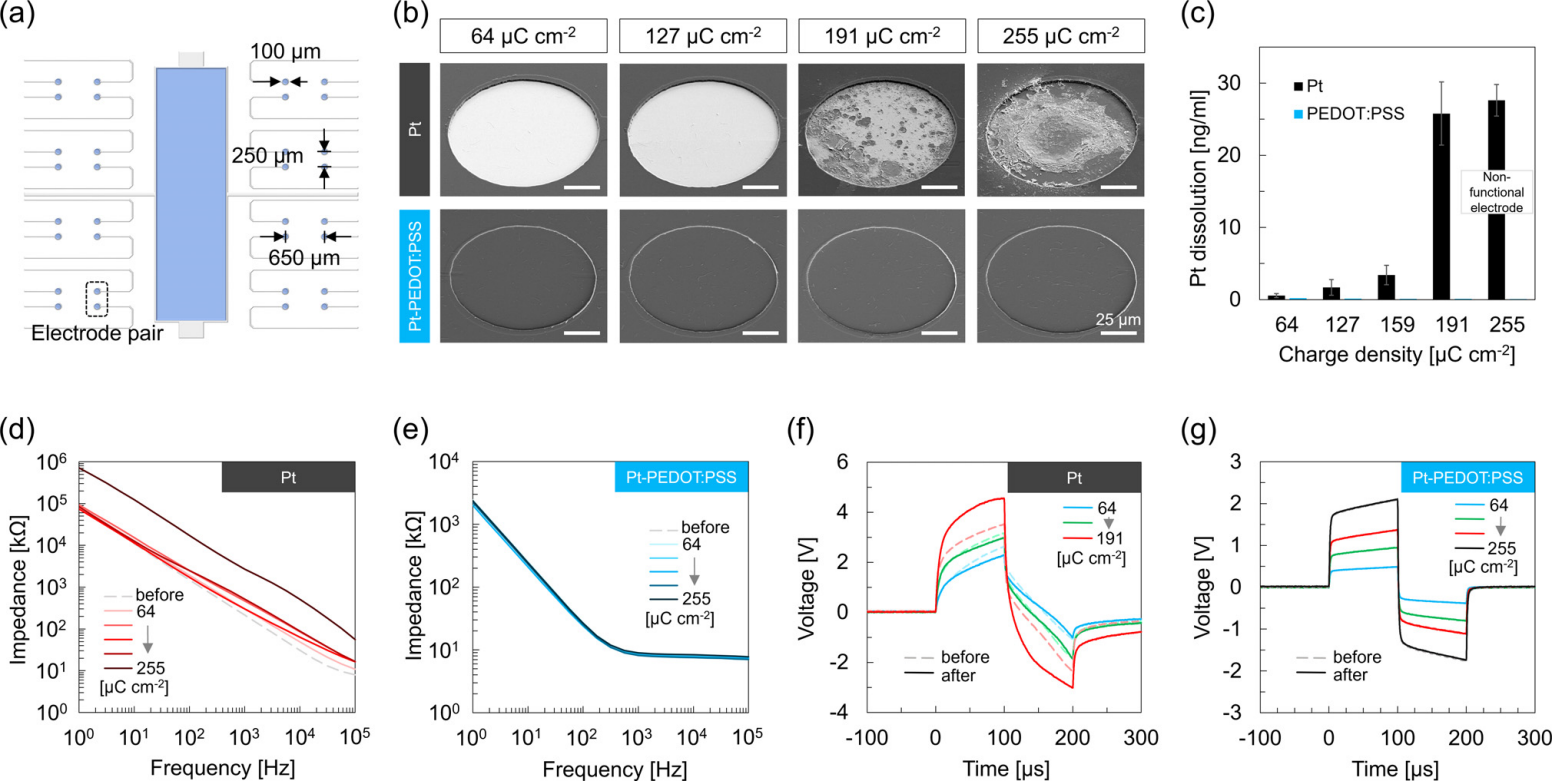
Pt and Pt-PEDOT:PSS stimulation performance. Electrodes were stimulated for 2 h with current-controlled, biphasic, 100 *μ*s per phase pulses at a frequency of 130 Hz with various charge densities. (a) Microelectrode configuration with 16 electrode pairs and a large electrode in the center that was not used in this work. (b) SEM images of the electrodes after stimulation. (c) Pt concentration in the electrolyte after stimulation. Values and error bars represent the average and standard deviation of three measurements, respectively. (d) and (e) Magnitude of the electrode impedance derived from EIS for Pt and Pt-PEDOT:PSS electrodes. (f) and (g) Voltage transient for Pt and Pt-PEDOT:PSS electrodes. (d)–(g) Data represents the average of three experiments for each charge density. Dashed lines indicate measurements before stimulation, and solid lines after stimulation. In (f), the voltage for 255 *μ*C cm^−2^ is not shown since the electrodes became nonfunctional after ∼1.5 h. In (g), the dashed lines overlap with the solid lines.

Microelectrodes were fabricated on a glass substrate and equipped with a PDMS well to confine culture media and cells (Fig. S1). The electrode array contained 32 electrodes with a diameter of 100 *μ*m which were configured in 16 pairs [Fig. 1(a)]. The distance between a pair of electrodes was 250 *μ*m and the distance between electrode pairs was at least 650 *μ*m. Two types of devices were fabricated, namely, bare Pt electrodes and PEDOT:PSS-coated Pt electrodes (Pt-PEDOT:PSS). The PEDOT:PSS coating had a thickness of 550 nm, which is optimal in terms of charge injection and stability.^52^ All experiments in this work were performed by applying current-controlled, biphasic, 100 *μ*s per phase pulses between a pair of electrodes.

### B. Electrode stability

The electrode stability was examined by monitoring visual changes, Pt dissolution, change in material composition, as well as change in EIS and voltage transient after 2 h of continuous stimulation in PBS. Figures 1(b) and S2 show SEM and optical images of Pt and Pt-PEDOT:PSS electrodes after stimulation with charge densities ranging between 64 and 255 *μ*C cm^−2^ per phase. Figure 1(c) shows the amount of Pt in the electrolyte after stimulation, which represents dissolved metal from the electrode. Pt electrodes showed changes in morphology and clear signs of degradation for ≥191 *μ*C cm^−2^. However, traces of Pt were found for the complete range of charge densities. Pt dissolution gradually increased as a function of charge density up to 159 *μ*C cm^−2^ after which it accelerated substantially. For a charge density of 255 *μ*C cm^−2^, the electrode became nonfunctional after ∼1.5 h of pulsing, i.e., the pulse generator reached its maximum potential and could no longer deliver 200 *μ*A pulses, indicating that the electrodes lost the ability to inject the desired charge. Although most of the Pt of the nonfunctional electrodes was released, some Pt was still observed on the glass substrate (but electrical connection with the interconnect was lost). Note that besides charge density, Pt dissolution also increases as a function of time.^27^ Therefore, prolonged stimulation with 64 *μ*C cm^−2^ is expected to eventually result in complete dissolution of the electrode. On the contrary, Pt-PEDOT:PSS electrodes showed no visual damage and no measurable traces of Pt up to 255 *μ*C cm^−2^.

Element analysis with EDX identified a change in composition of the Pt electrodes after stimulation with charge densities ≥191 *μ*C cm^−2^ (Fig. S3). Lower Pt contents confirmed the corrosion process, whereas higher contents of silicon and oxygen suggest that the underlying glass substrate got exposed at locations where Pt was completely dissolved. On the contrary, no change in elements was observed for Pt-PEDOT:PSS electrodes indicating the same surface composition after stimulation.

The electrochemical performance of both types of electrodes was examined with EIS and voltage transient before and after stimulation [Figs. 1(d)–1(g) and S4]. Stimulation with Pt electrodes increased the impedances for the complete range of charge densities with higher electrode impedances for higher charge densities. For 64–191 *μ*C cm^−2^, the increase occurred predominantly at frequencies >100 Hz, whereas for 255 *μ*C cm^−2^, the impedance increased by an order of magnitude over the complete range of frequencies. Pt electrodes that were pulsed with the lower charge densities (64 and 127 *μ*C cm^−2^) showed a minor decrease in voltage transient. However, for 191 *μ*C cm^−2^, the voltage transient increased and higher potentials were needed to inject the same amount of charge and, as discussed above, for 255 *μ*C cm^−2^, the Pt electrode became nonfunctional. Pt-PEDOT:PSS electrodes, compared to Pt electrodes, exhibited significant lower impedances and displayed lower voltage transients due to the high capacitance of PEDOT:PSS. Importantly, nearly no change in impedance and voltage transient were observed after stimulation for the complete range of charge densities. The impedance and voltage transient measurements illustrate increasingly more loss in electrochemical performance for higher current densities for the Pt electrodes, whereas Pt-PEDOT: PSS electrodes maintained their low impedance and charge injection performance.

### C. Neural stimulation and cell survival

The response of cortical neurons to stimulation pulses with the two different types of electrodes was examined with four types of experiments. The cell cultures on the devices were derived from rat brain cortices and formed dense clusters of cells, so-called neurospheres, that contained both neurons and astrocytes.

#### 1. Stimulation-induced activation of neurons

**FIG. 2.**
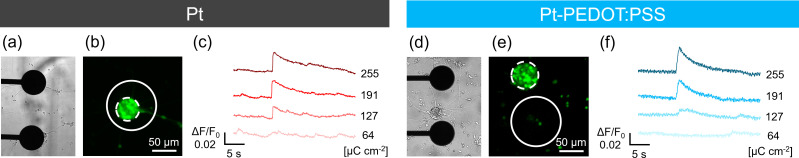
Acute activation of neurospheres. Fluorescent calcium responses were determined by applying 10 pulses for various charge densities. (a) and (d) Brightfield images of a pair of stimulation electrodes with cells and neurospheres. (b) and (e) The corresponding fluorescence images of activated neurospheres. The electrodes and neurospheres are indicated with solid and dashed white circles, respectively. Note that for (a) the neurosphere is located on top of the electrode and is not visible in the brightfield image. (c) and (f) Time traces of the normalized calcium signals from the neurosphere shown in (b) and (e).

Activation of neurons was examined for various charge densities using the fluorescent calcium indicator Fluo-4-AM. Figure 2 shows bright field images of the neurospheres and the electrode pairs that were used to stimulate, as well as representative stimulation evoked fluorescent calcium responses for Pt and Pt-PEDOT:PSS electrodes. Note that for the Pt electrode pair the neurosphere was located on top of one of the electrodes, whereas for the Pt-PEDOT:PSS pair, it was in between the electrodes. Pt and Pt-PEDOT:PSS electrodes showed similar performance in terms of stimulation-induced activation of cells as a function of charge density [Figs. 2(c) and 2(f)]. For both types of electrodes, calcium responses were observed for charge densities ≥127 *μ*C cm^−2^. Increasing the charge density led to activation of more cells in the neurospheres and for 191 *μ*C cm^−2^ almost the entire neurosphere was activated.

The comparable threshold shows that the charge density is an essential parameter for acute stimulation of cells for both types of electrodes. This implies that the distinct electrochemical properties of the two materials that cause differences in, for example, the electrode capacitance, Pt dissolution, and other faradaic reactions, have no direct effect on the acute activation of neurons.

#### 2. Cell survival after 2 h of continuous stimulation

Cell survival was assessed after the cells were subjected to 2 h of continuous stimulation with 191 *μ*C cm^−2^, which was the charge density at which Pt dissolution accelerated and the majority of the cells in the neurospheres were stimulated.

**FIG. 3.**
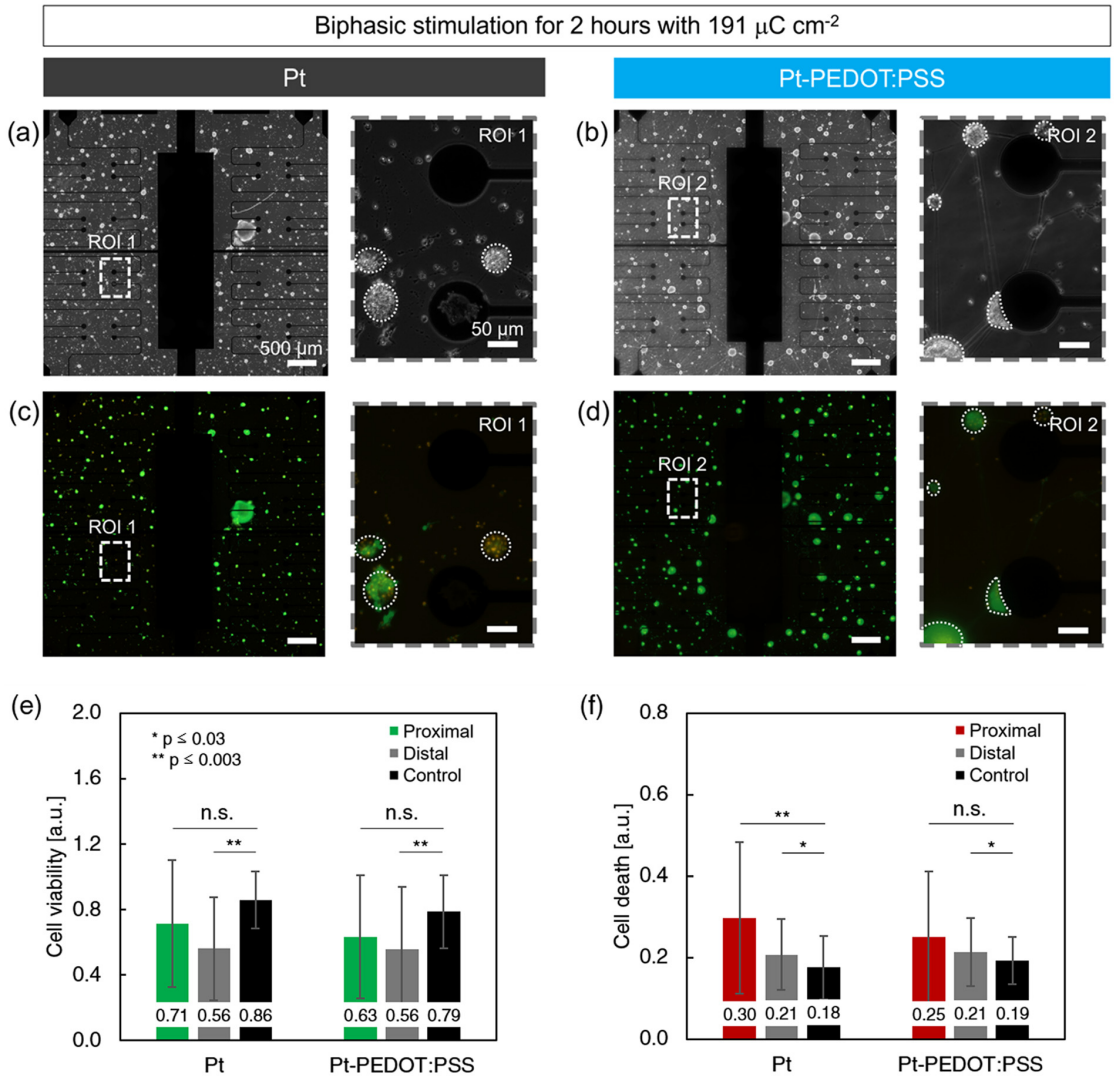
Cell survival after 2 h of stimulation with a charge density of 191 *μ*C cm^−2^. (a) and (b) Brightfield images of cell cultures on Pt and Pt-PEDOT:PSS electrodes, respectively. The entire electrode array is shown on the left and a zoom-in of the electrode pair used for stimulation on the right. (c) and (d) Corresponding fluorescence images of the bright field images showing calcein (green, live cells) and PI (red, death cells) staining. (e) and (f) Cell viability and cell death for proximal and distal neurospheres. Quantification (mean **±** s.d.) is based on calcein and propidium iodide (PI) fluorescence per neurosphere (two-sided paired t-test, with ^*^ p ≤ 0.03 and ^**^p ≤ 0.003). For this analysis, n = 298 neuropsheres on 14 devices (calcein) and n = 317 neuropsheres on 17 devices (PI) were analyzed from five separate stimulation experiments.

Figures 3(a) and 3(b) show brightfield images of the cultures across the entire microelectrode array (left) and 20× close-ups of electrode pairs used to apply electrical pulses (right). Neurospheres with diameters ranging from 30 to 95 *μ*m, were uniformly distributed across the entire device surface. The cultures also contained individual cells located in between the neurospheres as well as interconnections connecting all the cells and neurospheres together. The degradation of Pt electrodes manifested as bright, irregular shaped spots as was observed on seven out of nine electrode pairs [see the bottom electrode in ROI1 of Fig. 3(a)]. Pt-PEDOT:PSS electrodes did not show any changes in the brightfield images (0/14 pairs).

Figures 3(c) and 3(d) show the corresponding fluorescence images of live cells in green (calcein) and dead cells in red (PI). Calcein within live cells is distributed throughout the cytosol causing the complete cell to become fluorescent. Therefore, neurospheres containing primarily living cells displayed bright and green, covering nearly the complete surface area of the neurosphere. Individual cells within a neurosphere could not always be distinguished in the fluorescent signals due to the 3D organization of cells in the neurospheres. PI only stains the cell nuclei such that clusters with high levels of dead cells were less bright and showed more red dots within the neurospheres.

Cell viability and cell death were quantified by determining the area for each fluorescent indicator per neurosphere [Figs. 3(e) and 3(f)]. The control experiments show the values for cells that were not stimulated. Although we carefully developed protocols for healthy and reproducible cell cultures, dead cells were found in the center of nearly all neurospheres. This was observed in the controls with cell viability values below 1 (Pt: 0.86, Pt-PEDOT:PSS: 0.79) and cell death values above 0 (Pt: 0.18, Pt-PEDOT:PSS: 0.19). Nonetheless, high levels of live cells indicated that the presence of the electrode materials had no severe effect on cell survival.

For the stimulation experiments, neurospheres located proximal and distal from the stimulation electrodes were categorized separately. For each type of electrode, two-sided t-tests with respect to the control were performed to determine statistically significant differences. For distal neurospheres, no difference was found between the Pt and Pt-PEDOT:PSS electrodes. Both types of electrodes showed reduced cell viability (both 0.56, p ≤ 0.003) and increased cell death (both 0.21, p ≤ 0.03) indicating that the electrical stimulation affected the survival of cells located more than 500 *μ*m from the electrode pair. For proximal neurospheres, no significant difference was found in cell viability (Pt: 0.71 and Pt-PEDOT:PSS: 0.63, both n.s.). However, significant higher levels of cell death were found for the Pt electrodes which were not found for Pt-PEDOT:PSS electrodes (Pt: 0.30, p ≤ 0.003 and Pt-PEDOT:PSS: 0.25, n.s.). Overall, Pt electrodes showed reduced cell survival for both proximal and distal neurospheres, whereas Pt-PEDOT:PSS electrodes only affected distal neurospheres.

#### 3. Cell survival after 2 h of continuous stimulation on three consecutive days

**FIG. 4.**
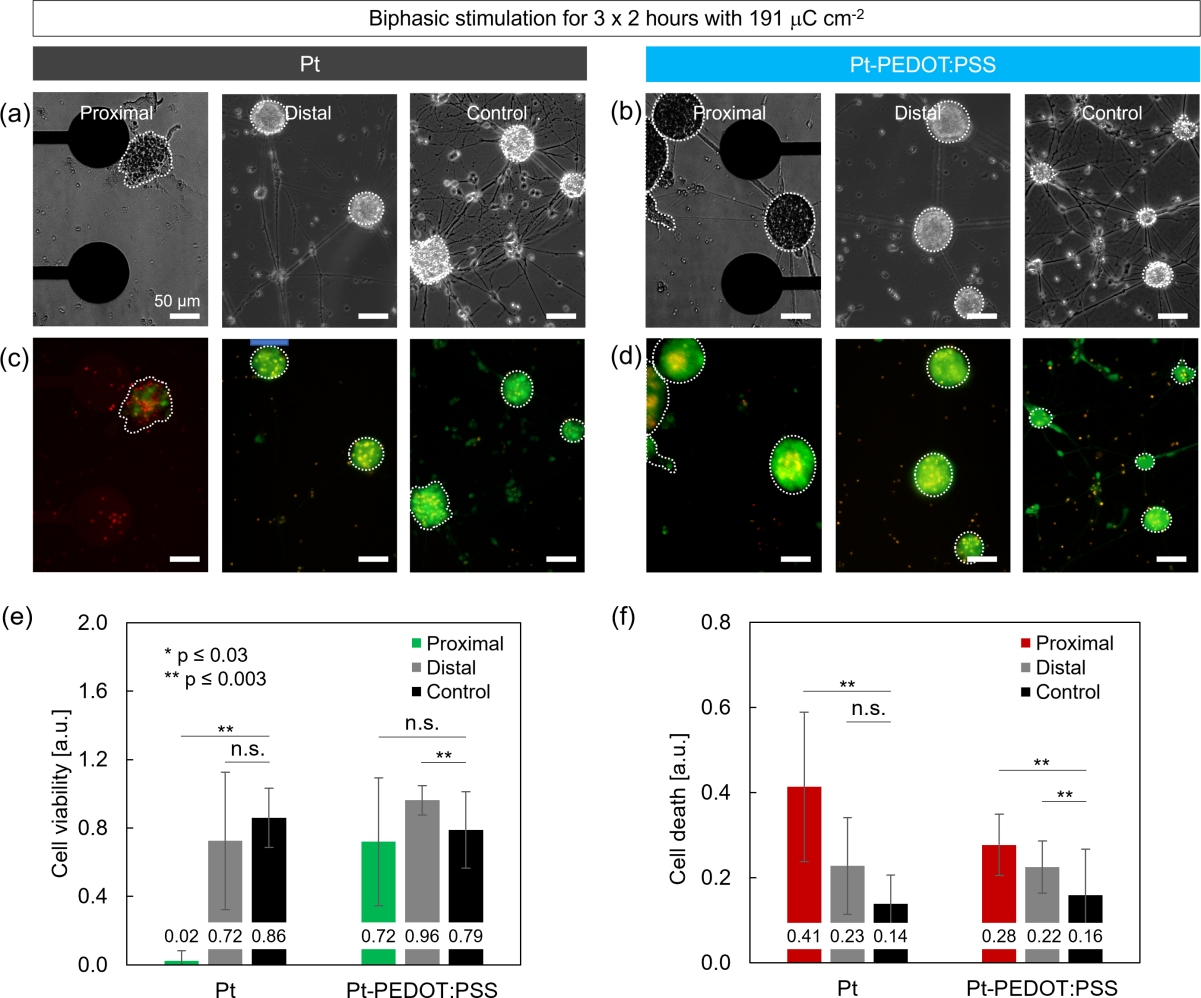
Cell survival after 2 h of stimulation on three consecutive days with a charge density of 191 *μ*C cm^−2^. (a) and (b) Brightfield images and (c) and (d) corresponding live/dead fluorescent images of proximal and distal neurospheres on Pt and Pt-PEDOT:PSS electrodes and their controls. (e) and (f) Cell viability and cell death for Pt and Pt-PEDOT:PSS electrodes. Quantification (mean **±** s.d.) is based on calcein and propidium iodide (PI) fluorescence per neurosphere (two-sided paired t-test, with ^*^p ≤ 0.03 and ^**^p ≤ 0.003). For this analysis, n = 184 neurospheres on six devices (calcein) and n = 106 neurospheres on six devices (PI) were analyzed from two separate stimulation experiments.

To examine the influence of repetitive stimulation on cell survival, the 2-h stimulation protocol was applied for three consecutive days. Figures 4(a)–4(d) show brightfield and fluorescence images of proximal and distal neurospheres from the electrodes and their controls. Remarkably, while most neurospheres showed abundant presence of green and, thus, alive cells, nearly all proximal neurospheres on Pt electrodes showed only marginal calcein fluorescence and abundant PI florescence. The quantified cell viability (0.02, p ≤ 0.003) and cell death (0.41, p ≤ 0.003) confirmed that almost no cells survived the stimulation [Figs. 4(e) and 4(f)]. Note that cell death is quantified by calculating the ratio of the PI fluorescence area and the neurosphere area. This number is expected to be substantially lower than 1 because PI stains the cell nuclei which only occupies a portion of the cytoplasm. Indeed, Pt electrodes where nearly all proximal cells died showed a mean value of 0.41 for cell death. On the contrary, Pt-PEDOT:PSS electrodes showed no significant change in the cell viability (0.72, n.s.) and despite increased levels of cell death (0.28, p ≤ 0.003), these live/dead values indicate that a considerable part of the neurospheres had survived. For distal neurospheres on Pt electrodes, cell viability (0.72, n.s.) and cell death (0.23, n.s.) were not affected after 3 days of stimulation. Distal neurospheres on Pt-PEDOT:PSS electrodes showed a surprisingly high cell viability (0.96, p ≤ 0.003), while also showing an increase in cell death (0.22, p ≤ 0.003).

Overall, Pt electrodes showed extremely low cell survival for proximal neurospheres and no changes for distal neurospheres demonstrating local effects that are severe and harmful. Stimulation-induced changes in cell survival for Pt-PEDOT:PSS electrodes were less pronounced. However, both proximal and distal neurospheres were affected.

#### 4. Cell survival by exposing cells to conditioned media

**FIG. 5.**
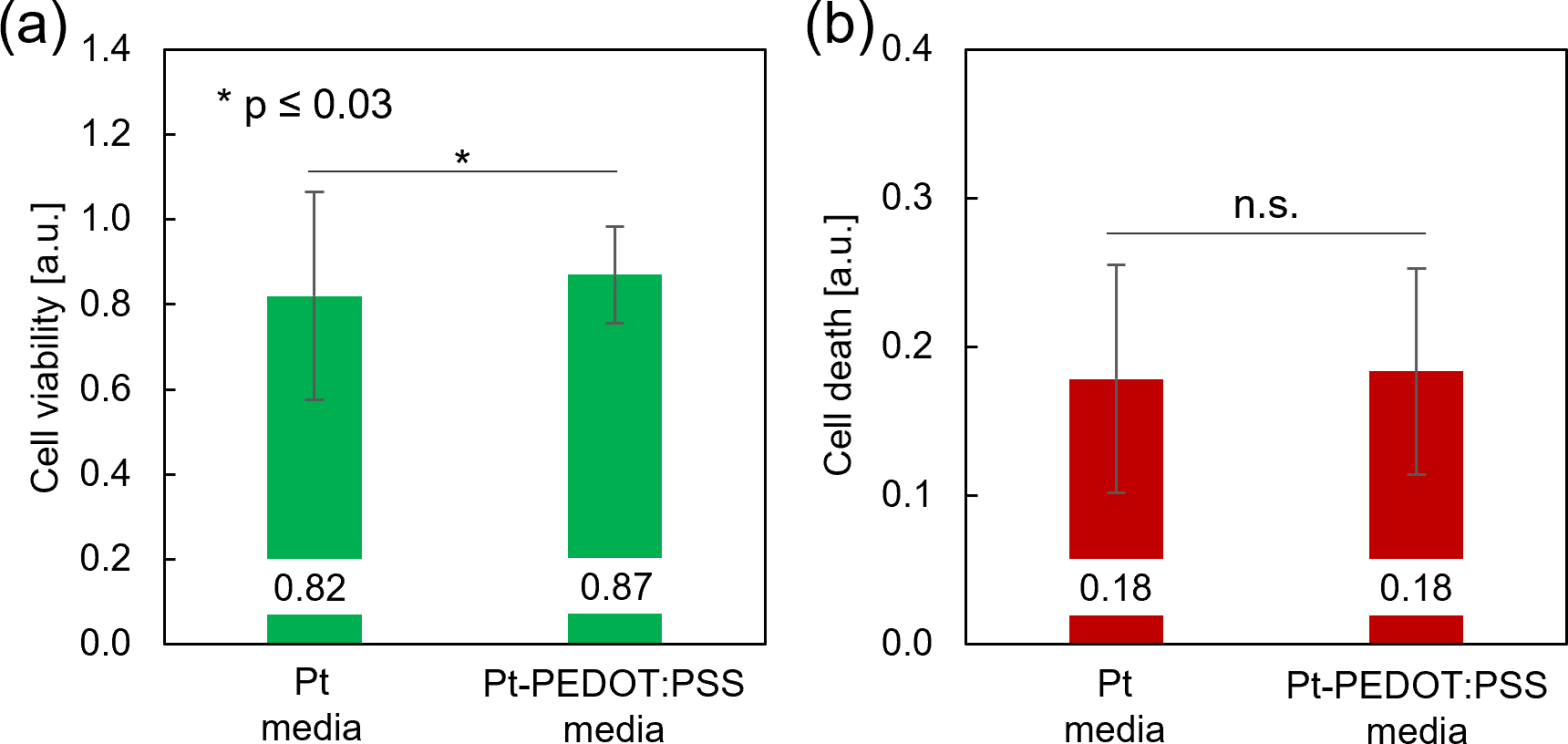
Cell survival after 3 days in conditioned media that was obtained after 2 h of stimulation with a charge density of 191 *μ*C cm^−2^ on Pt and Pt-PEDOT:PSS devices. (a) and (b) Cell viability and cell death for Pt and Pt-PEDOT:PSS electrodes. Quantification (mean **±** s.d.) is based on calcein and PI fluorescence per neurosphere. For this analysis, n = 158 neurospheres (calcein) and n = 163 neurospheres (PI) were evaluated on four devices from one experiment (two-sided paired t-test, with ^*^p ≤ 0.03).

To isolate the effect of dissolved Pt from the application of electrical pulses, cell viability and cell death of cortical cultures were evaluated in so-called “conditioned media,” i.e., media that was obtained by pulsing the electrodes without cells. After 3 days in conditioned media, slightly higher levels of cell viability were found in Pt-PEDOT:PSS conditioned media [Fig. 5(a)]. However, no difference in cell death was observed between the conditioned media [Fig. 5(b)].

## III. DISCUSSION

### A. Threshold at which Pt dissolution accelerates

Pt dissolution was determined for current densities of 64–255 *μ*C cm^−2^ because this is the range where we observed activation of cells in the neurospheres. The bare Pt electrodes showed stimulation-induced release of Pt that increased non-linearly as a function of charge density and accelerated at values ≥191 *μ*C cm^−2^. Kumsa *et al.* reported acceleration of Pt dissolution around 80 *μ*C cm^−2^ for 1 mm diameter electrodes^21^ or more generally at values where k > 1.75,^53^ where k is defined by the Shannon equation.^19^ In our study, 191 *μ*C cm^−2^ corresponds to k = 0.46 (I = 150 *μ*A, t = 100 *μ*s, and electrode d = 50 *μ*m) which means we found higher charge densities, but lower values of k. However, direct comparison of the results is not possible due to important differences in the experimental setup. We used a bipolar electrode configuration and did not include a Ag/AgCl reference and carbon counter electrode. We also employed a different pulse generator and applied a symmetric biphasic pulse instead of an asymmetric biphasic pulse (with capacitive discharge) with 100 *μ*s inter-phase delay. A study by Shepherd *et al.* reported significantly increase in Pt electrode corrosion at charge densities 267 *μ*C cm^−2^; however, this was in the cochlea of guinea pigs in a tripolar electrode configuration.^24^ Despite these discrepancies, Pt dissolution on neural stimulation electrodes, as already reported in 1977 by Brummer *et al.,*^20^ was confirmed for thin film electrodes in a bipolar configuration.

### B. Improved electrode integrity

The Pt electrodes subjected to charge densities ≥191 *μ*C cm^−2^ for 2 h showed significant damage and loss in charge injection performance due to corrosion. For lower charge densities, we also found Pt dissolution, which indicates that prolonged stimulation will consume the entire electrode eventually. Such material deterioration limits the lifetime of the electrode which becomes particularly important for thin film devices with metallic layers of only a few 100 nm thick. The development of stable, thin film electrode materials is essential to realize flexible and conformal implantable electrode arrays with reduced thickness that have shown to be steadier and more reliable.^54^

This work demonstrates a stable PEDOT:PSS coating that prevents Pt dissolution; however, we did not investigate what mechanism prevents the dissolution. The volumetric capacitive property of PEDOT:PSS suggests that most of the electrode–electrolyte interaction occurs within the bulk of the polymeric material.^55^ It is unclear to what degree the electrolytic components reach Pt and what the nature is of the possible interactions. Reduced transient voltages limits the electrode potentials, which might prevent activation of the dissolution reactions.

The PEDOT:PSS coating extends the electrode lifetime; however, we did not examine the thresholds at which the coating degrades. Increasing the current density and/or prolonged stimulation might eventually lead to loss of performance. Previous studies have shown failure of PEDOT:PSS electrodes for higher charge densities^52^ and accelerated aging conditions^39^ in the form of delamination and cracking. We observed no visual damage, no Pt dissolution, and no change in the electrochemical performance indicating that the coating is intact and functional. The results presented here show that PEDOT:PSS improves the durability of Pt electrodes, which makes conducting polymers a promising candidate for a long-term neural stimulation interface and motivates further investigations.

### C. Influence of the experimental setup on the electrode stability

The first part of this work was performed without cells in PBS at room temperature for 2 h, whereas the second set of experiments were performed with cells in cell media at 37 °C for 7–10 days. These differences had an important effect on the performance of the electrodes. Pt electrodes were damaged in both cases but seemed to corrode less on devices containing cells compared to the devices in PBS. This is most likely related to organic contaminants such as proteins that significantly inhibit Pt dissolution and, consequently, reduce electrode corrosion.^25^ None of the 15 Pt-PEDOT:PSS electrode pairs showed any signs of damage after pulsing in PBS. However, four Pt-PEDOT:PSS electrode pairs showed Pt-like voltage transients after incubation with cells prior to stimulation. Five electrode pairs failed during the stimulation of cells showing Pt-like voltage transients suggesting that the PEDOT:PSS coating delaminated. Three Pt electrode pairs became nonfunctional during the stimulation of cells. Electrodes that showed such abnormal performance were not included in the analysis. Further investigation is needed to study the influence of parameters, such as electrolyte, temperature, presence of cells, and duration on the stability PEDOT:PSS-coated electrodes.

### D. Avoiding Pt dissolution

Charge-imbalanced waveforms have shown to mitigate the dissolution of Pt electrodes by reducing the anodic potential excursion and therewith Pt oxidation and dissolution.^15,21,56^ Electrode shorting with coupling capacitors which reduces residual DC levels *in vivo* is another strategy to reduce Pt dissolution.^57^ Such strategies allow more charge injection with less Pt loss, however, do not completely eliminate Pt dissolution. Coating Pt electrodes with a graphene monolayer greatly reduces dissolution by acting as a diffusion barrier; however, the robustness of such coating is questioned since some Pt was found in the electrolyte.^27^

On the contrary, PEDOT:PSS-coated electrodes showed no measurable Pt dissolution with ICP-MS or changes with other characterization methods (SEM, EDX, EIS, and voltage transient). Pt or PEDOT:PSS dissolution might have occurred at a slow rate; however, any significant changes in the electrochemical surface area would have been observed in the EIS. Interestingly, coating gold electrodes with PEDOT:PSS does not completely prevent gold dissolution.^39^ Indeed, gold dissolves at a faster rate than Pt,^58^ indicating that the underlying metallic material is an important point of consideration.

### E. Limitations of 2D imaging of 3D neurospheres

Cell survival was assessed through both cell viability with calcein and cell death with PI. Both assays were used because the complex organization of cells in neurospheres hinders optical access. Our method projects a 3D cell culture on a 2D imaging plane and determines the surface area of fluorescence in a neurosphere. Cells that are stacked in the vertical direction overlap on the fluorescent image and are not distinguished such that the cell viability is only affected if all the cells in the vertical direction showed decreased levels of calcein fluorescence. Similarly, PI positive cells that are located on top of each other are not individually identified. Nonetheless, these fluorescent assays form a well-established live/dead staining and provide practical means to assess cell survival.

### F. Cell survival after electrical stimulation

We examined the effect of neural stimulation with two electrode materials that exhibit fundamental different electrochemical properties. Cell survival was assessed with a charge density of 191 *μ*C cm^−2^ per phase because this charge density was associated with the activation of the neurons on both Pt and Pt-PEDOT:PSS electrodes. Moreover, significant dissolution of Pt electrodes was found for this threshold, while not dissolving the entire electrode.

Electrical stimulation with Pt electrodes had a severe effect on cell survival located close to the electrodes. This was observed in the increased cell death after 2 h of stimulation, an effect that enhanced for 3 × 2 h of stimulation where nearly all cells died. Pt-PEDOT:PSS electrodes showed no significant effect on proximal cell survival after 2 h of stimulation. Extending the stimulation time to 3 × 2 h killed a significant number of cells; however, high levels of viability indicated considerable survival of the proximal neurospheres. This result stresses the influence of electrode material and demonstrates that PEDOT:PSS coatings reduce stimulation-induced cell damage.

Pt electrodes also reduced distal cell survival after 2 h of stimulation. Reduced viability and increased death were found after 3 × 2 h of stimulation, but these were not statistically significant. Pt-PEDOT:PSS electrodes showed a similar trend indicating that stimulation with both electrode materials affected survival of neurospheres located more than 500 *μ*m from the electrodes.

Current-controlled pulses ensured that the difference between Pt and Pt-PEDOT:PSS electrodes was not caused by the charge density. However, the charge injection distribution might play a role. Zheng *et al.* suggested that the electrode materials affect the electric field distribution, where nonuniformity will increase the activation of nearby neural elements.^59^ The electrode material also plays a crucial role in the electron transfer processes that occur at the interface. For example, Ehlich *et al.* showed that the nature of oxygen reduction reactions and the possible generation of hypoxic conditions and/or toxic hydrogen peroxide is electrode material dependent.^45^ Further studies are needed to investigate how these differences influence biological responses.

Alternatively, Pt dissolution is proposed as one of the noxious byproducts of electrical stimulation. Mortimer and co-workers have shown that tissue damage, as predicted by Shannon, occurs at charge injection thresholds where Pt dissolution increases significantly.^60^ Moreover, Pt particles and compounds have shown to induce cell death.^23^ In this work, toxicity of dissolved Pt was likely not the only cause of reduced cell survival because Pt-PEDOT:PSS electrodes did not release measurable amounts of Pt, yet induced some levels of cell damage. We found some reduced cell viability with Pt conditioned media but this was not sufficient to explain the observations with stimulated cells. For Pt to be toxic for cells, concentrations must exceed certain thresholds.^23^ The toxicity threshold is three orders of magnitude larger than the dissolved Pt we determined indicating that thin film electrodes do not contain enough Pt to have an adverse effect on cells. An important difference between the cell stimulation and the conditioned media experiments is distribution of Pt throughout the electrolyte. Due to processing, the dissolved Pt in the conditioned media was homogenously mixed throughout the media. Cells during the stimulation experiments were likely exposed to higher concentrations because of their vicinity to the electrodes where the dissolution is occurring. Characterization of Pt diffusion and distribution throughout the media would give a better insight into local concentrations.

Prolonged electrical stimulation above certain safe thresholds lead to tissue damage, which is described by the Shannon equation.^19^ These limits are based on empirical, histological data and do not describe mechanisms that cause tissue damage. Various mechanisms have been hypothesized including neural hyperactivity (overstimulation) and toxic species from faradaic reactions at the electrode interface.^15,61^ Further investigation is imperative to fully unravel the mechanism of tissue damage caused by electrical stimulation. This work demonstrates the influence of the electrode material and higher levels of cell survival with PEDOT:PSS-coated electrodes, which encourages further characterization toward its safety for neural stimulation devices.

## IV. CONCLUSION

This work compared the neural stimulation performance of Pt and Pt-PEDOT:PSS electrodes in a bipolar configuration. Stability analysis revealed that Pt electrodes corrode and major electrode damage was found for charge densities ≥191 *μ*C cm^−2^. On the contrary, the organic coating did not show any signs of degradation and prevented Pt dissolution, demonstrating a durable electrode that is more suitable for chronic applications. Both electrode types induced neural activation upon stimulation with charge densities above 127 *μ*C cm^−2^. Larger charge densities resulted in more activation of neurons within the neurospheres. After 2 h of stimulation, higher levels of cell survival were found for Pt-PEDOT:PSS electrodes. Stimulation with Pt electrodes predominantly affected cells that were in the vicinity of the electrodes. Longer stimulation times increased the cell death for both electrodes but was most fatal for Pt electrodes. This work shows the benefit of PEDOT:PSS coatings for Pt electrodes by significantly improving the electrode lifetime as well as protecting cells at the electrode–tissue interface during neural stimulation.

## V. METHODS

### A. Device fabrication

Standard thin-film microfabrication processes, based on previously reported protocols, were used for the fabrication of the *in vitro* devices.^50,51^ The process included patterning of Pt, device encapsulation, and deposition of the PEDOT:PSS coating. The Pt electrodes and leads were patterned with a liftoff process. A positive photoresist S1813 layer (1.5 *μ*m) was spin-coated, followed by exposure with an MBJ4 contact aligner and development in MF-26A. Titanium (10 nm) and Pt (120 nm) were deposited with an Alliance Concept EVA 450 electron beam evaporator. Pt was deposited at 0.5 Å/s and in eight steps of 15 nm with 15 min of waiting time between each step to avoid cracking of the photoresist. Liftoff was performed in acetone. To promote the adhesion of the encapsulation layer, the devices were treated with an oxygen plasma and 3-(trimethoxysilyl)propyl methacrylate (A-174 silane) was applied when depositing a 2 *μ*m layer of Parylene C with a Specialty Coating Systems PDS 2010. Coating the electrodes with PEDOT:PSS was achieved by using a dry liftoff technique employing a sacrificial Parylene C layer as follows. After spin coating an anti-adhesion layer of diluted micro-90 detergent, a 2 *μ*m sacrificial layer of Parylene C was deposited. To open the electrodes and connection pads, positive photoresist AZ10XT was spin coated, exposed, and developed with AZ developer followed by reactive ion etching using an Oxford 80 Plasmalab plus. PEDOT:PSS was deposited by spin-coating a mixture of Clevios PH1000, glycerol, dodecyl benzene sulfonic acid, and (3-glycidyloxypropyl)trimethoxysilane (Sigma Aldrich). To achieve a final thickness of about 550 nm, four layers were spin-coated with baking at 110 °C for 1 min after each layer. The sacrificial Parylene C layer was manually peeled off, and the device was baked at 140 °C for 1 h and rinsed in de-ionized water for 1 h. For the Pt electrodes (without PEDOT:PSS coating), the same process was followed except for the deposition of the PEDOT:PSS mixture.

### B. Electrical stimulation

All electrical stimulation experiments were performed with the following setup and parameters. Electrical pulses were generated with an A-M systems model 4100, a programmable isolated pulse generator. A bipolar electrode configuration was used, i.e., the pulses were applied between two adjacent electrodes that we have called “an electrode pair.” The applied pulses were current-controlled, biphasic, and symmetric with a pulse duration of 100 *μ*s for each phase, 0 *μ*s inter-phase delay, and a repetition frequency of 130 Hz. Various stimulation amplitudes in the range of 50–200 *μ*A were used as specified in the sections below. To verify pulse delivery and monitor possible electrode degradation, the current and voltage were continuously monitored with a Rohde & Schwarz RTM3004 oscilloscope, a RT-ZC15B current probe, and a RT-ZP05S voltage probe.

### C. Electrode stability and Pt dissolution

Pulses were applied for a duration of 2 h (9.36 × 10^5^ pulses) to pairs of electrodes in 200 *μ*l phosphate buffered saline (PBS) with current amplitudes of 50, 100, 125, 150, and 200 *μ*A. For electrodes with a diameter of 100 *μ*m and a surface area of 7.85 × 10^−5^ cm^2^, these current amplitudes correspond to charge densities of 64, 127, 159, 191, and 255 *μ*C cm^−2^ per phase, respectively. Each charge density was measured three times and all experiments were performed with a new pair of electrodes. Electrode degradation was assessed electrochemically, visually and through Pt trace analysis. Electrochemical impedance spectroscopy (EIS) and voltage transient were measured before and after the pulses in fresh PBS. EIS was performed with a PalmSens4 Potentiostat in a two-electrode setup where the pair of electrodes functioned as the working and counter electrodes (frequency: 1–10^5^ Hz, voltage: 25 mV, four points per decade). The voltage transient was performed by recording the potential when a pulse was applied to a pair of electrodes. For the Pt trace analysis, the 200 *μ*l of PBS used for the pulsing was collected and an additional 200 *μ*l of PBS was used to rinse the electrodes. The total of 400 *μ*l was analyzed with inductively coupled plasma mass spectroscopy (ICP-MS). Prior to ICP-MS analysis, all samples were diluted five times with MilliQ water (18.2 MΩ cm obtained from a Direct-Q 5 Ultrapure water system, Merck Millipore, Massachusetts, USA). Quantification of Pt by ICP-MS was performed based on external calibration by measuring Pt standards in the concentration range of 0.01–100 *μ*g l^−1^ with online internal standardization (25 *μ*g l^−1^ solution of Ir). Calibration standard solutions were prepared from Pt stock solution (1000 mg l^−1^ in 8% HCl obtained from Merck, Darmstadt, Germany). For ICP-MS analysis, an Agilent 7700× (Agilent Technologies, Tokyo, Japan) was used. Optimization of instrumental parameters was performed daily to obtain the required sensitivity and low levels of oxides and doubly charged ions. Scanning electron micrographs (SEM) and element analysis by means of energy dispersive x-ray (EDX) were acquired with a Carl Zeiss Ultra 55.

### D. Cell response assessment

#### 1. Cell cultures

A custom-made polydimethylsiloxane (PDMS) well of 1–2 mm thick was attached on top of the device to confine cells within a 0.5 × 0.5 cm^2^ area during seeding and a manually cut 50 ml falcon tube was added on top to contain cell media during culture. Devices were plasma treated at 25 W for 1 min, sterilized with 70% ethanol for 30 min, and rinsed with Dulbecco's phosphate-buffered saline (DPBS, ThermoFisher). Cleaned devices were coated with 100 *μ*l of 50 *μ*g ml^−1^ poly-D-lysine (70 kDa, Sigma Aldrich) in DI water for 2 h at 37 °C, rinsed, and left overnight in DPBS at 37 °C. After a second coating with 100 *μ*l of 20 *μ*g ml^−1^ of laminin (Sigma Aldrich) in DPBS for 2 h at 37 °C, devices were rinsed and placed in the incubator with fresh DPBS until cell seeding. E15 rat cortical tissues (NeuroSys, France) were dissociated with 2 mg/ml papain solution of Hibernate E-Ca without B27 (Brainbits, LLC) for 10 min in a water bath at 30 °C, triturated in Hibernate E containing 2% B27 and 0.5 × 10^−3^ M Glutamax (Hibernate EB media, Brainbits, LLC) to disperse most of the tissue, spun at 200 G for 1 min, and resuspended in NeuroCult^TM^ Neuronal Plating Media (Stemcell Technology). Cells were plated at a high cell density of 900 cells/mm^2^ to enhance the electrical activity by creating neurospheres,^49^ incubated at 37 °C with 5% CO_2_, and cultured for the first 5 days in 4 ml of Neurocult^TM^ media containing 0.5 mM L-Glutamax (Gibco) and 25 *μ*M L-glutamic acid (Sigma Aldrich). Next, 2 ml of the media was removed and 3 ml of BrainPhys^TM^ Neuronal Media (Stemcell Technology) with 2% Neurocult^TM^ Neuronal supplement (Stemcell Technology) and 1% Penicillin–Streptomycin (ThermoFisher) was added. If cultures were kept for more than 7 days, half of the media was replaced by fresh media every 3 days after the initial media exchange.

#### 2. Cell stimulation to induce neural activity

On days *in vitro* (DIV) 6–9, cells were loaded with 1 mM Fluo-4-AM (ThermoFisher) for 45 min at 37 °C, followed by a single wash and 45 min of incubation to allow complete dye de-esterification. Pulsing was performed in a Faraday cage on a TC-324C temperature-controlled stage (Warner Instruments) set at 37 °C. One electrode pair was used to electrically stimulate cells by applying 10 biphasic pulses, as described above, with current amplitudes of 50, 100, 150, and 200 *μ*A, corresponding to charge densities of 64, 127, 191, and 255 *μ*C cm^−2^ per phase. Fluorescence images of intracellular Ca^2+^ signals were acquired using a Carl Zeiss microscope (20× objective, excitation at 470 nm). Images were processed and analyzed using WinFluor. Ca^2+^ signals are expressed as F-F_0_/F_0_, where F corresponds to the average fluorescence and F_0_ to the fluorescence signal of the first 30 s pre-stimulation. The biological origin of the fluorescence images was verified by the addition of ionomycin (Sigma, 1 mM in DMSO).

#### 3. Cell stimulation to assess cell survival

On DIV7-11, cells were electrically stimulated for 2 h with a current amplitude of 150 *μ*A (191 *μ*C cm^−2^ per phase) applied to one electrode pair. Stimulation was performed in a Faraday cage on a TC-324C temperature-controlled stage (Warner Instruments) set at 37 °C. Cell survival was evaluated by quantifying cell viability and cell death. Live/dead assays were performed 24 h after stimulation by incubation in fresh media with ∼0.2 *μ*g calcein-AM and 0.3 *μ*g Propidium Iodide (PI) for 10 min. Green fluorescent calcein was used to determine cell viability and red fluorescent PI to determine cell death. Fluorescence images were acquired using a Carl Zeiss inverted microscope (Axio Observer Z1).

#### 4. Control experiment with conditioned media

Cell media was “conditioned” by pulsing four electrode pairs without cortical cells for 2 h with a charge density of 191 *μ*C cm^−2^ on both Pt and Pt-PEDOT:PSS devices. The conditioned media was used to evaluate cell survival based solely on the possible change in media composition (as a results of electrochemical reactions) while excluding the effect of stimulating cells. Multiple pairs of electrodes were used to increase the effect of exposing cells to possibly more byproducts created in the conditioned media. The conditioned media was stored at 4 °C and used when exchanging media for cell cultures on DIV10. Cell survival assays were performed 3 days after the media exchange.

#### 5. Statistical analysis

To determine whether the distance between the cell and the electrode pair influences possible stimulation-induced cell damage, we analyzed cell viability and death for “proximal” (defined as region of interest (ROIs) of 300 × 400 *μ*m^2^ around the electrode pair) and “distal” (defined as ROIs located >500 *μ*m from the electrode pair) cells. Images were analyzed using CellProfiler Software, which allows for unbiased and automated detection of live and dead cells within the neurospheres. Cell viability was quantified by dividing the area of green fluorescent calcein within a neurosphere by the total surface area of the neurosphere. The same calculation was applied for the PI fluorescence to quantify cell death. The total surface area of a neurosphere was determined with the brightfield images using the Global Otsu threshold measure of CellProfiler. Using the fluorescent images, the calcein area was determined with the global Otsu threshold measure and the PI area with the global minimum cross-entropy method. This method resulted in a quantification of cell viability and cell death per neurosphere.

A statistical analysis was performed with a paired two-sided t-test. The staining values per neurosphere were categorized per distance from the stimulation pair, i.e., proximal or distal, and compared to the controls (not stimulated neurospheres) per device type. For cell survival after 2 h of stimulation with a charge density of 191 *μ*C cm^−2^, a total of n = 298 neurospheres were analyzed on 14 devices for cell viability and n = 317 neurospheres on 17 devices for cell death from five separate stimulation experiments. For cell survival after 2 h of stimulation on three consecutive days, a total of n = 184 neurospheres were analyzed on six devices for cell viability and n = 106 neurospheres on six devices for cell death from two separate stimulation experiments. An overview of the resulting p-values is provided in Table S1.

## SUPPLEMENTARY MATERIAL

See the supplementary material for details: Microelectrode array (Fig. S1), optical images of Pt and PtPEDOT:PSS electrodes after 2 h of stimulation (Fig. S2), element analysis before and after 2 h of stimulation (Fig. S3), phase of the EIS before and after 2 h of stimulation (Fig. S4), and p-values of statistical analysis on cell viability and cell death (Table S1).

## Data Availability

The data that support the findings of this study are available from the corresponding author upon reasonable request.
